# Abnormal placental cord insertion and adverse pregnancy outcomes: a systematic review and meta-analysis

**DOI:** 10.1186/s13643-017-0641-1

**Published:** 2017-12-06

**Authors:** Khadijah Irfah Ismail, Ailish Hannigan, Keelin O’Donoghue, Amanda Cotter

**Affiliations:** 1 Obstetrics and Gynaecology Department, Graduate Entry Medical School, University of Limerick, Limerick, Ireland; 2 Biostatistics Department, Graduate Entry Medical School, University of Limerick, Limerick, Ireland; 3 Obstetrics and Gynaecology Department, University College Cork, Cork, Ireland

**Keywords:** Abnormal placental cord insertion, Marginal placental cord insertion, Velamentous placental cord insertion, Adverse pregnancy outcomes, Small for gestational age, Emergency cesarean delivery

## Abstract

**Background:**

Abnormal placental cord insertion (PCI) includes marginal cord insertion (MCI) and velamentous cord insertion (VCI). VCI has been shown to be associated with adverse pregnancy outcomes. This systematic review and meta-analysis aims to determine the association of abnormal PCI and adverse pregnancy outcomes.

**Methods:**

Embase, Medline, CINAHL, Scopus, Web of Science, ClinicalTrials.gov, and Cochrane Databases were searched in December 2016 (from inception to December 2016). The reference lists of eligible studies were scrutinized to identify further studies. Potentially eligible studies were reviewed by two authors independently using the following inclusion criteria: singleton pregnancies, velamentous cord insertion, marginal cord insertion, and pregnancy outcomes. Case reports and series were excluded. The methodological quality of the included studies was assessed using the Newcastle-Ottawa Scale. Outcomes for meta-analysis were dichotomous and results are presented as summary risk ratios with 95% confidence intervals.

**Results:**

Seventeen studies were included in the systematic review, all of which were assessed as good quality. Normal PCI and MCI were grouped together as non-VCI and compared with VCI in seven studies. Four studies compared MCI, VCI, and normal PCI separately. Two other studies compared MCI with normal PCI, and VCI was excluded from their analysis. Studies in this systematic review reported an association between abnormal PCI, defined differently across studies, with preterm birth, small for gestational age (SGA), low birthweight (< 2500 g), emergency cesarean delivery, and intrauterine fetal death. Four cohort studies comparing MCI, VCI, and normal PCI separately were included in a meta-analysis resulting in a statistically significant increased risk of emergency cesarean delivery for VCI (pooled RR 2.86, 95% CI 1.56–5.22, *P* = 0.0006) and abnormal PCI (pooled RR 1.77, 95% CI 1.33–2.36, *P* < 0.0001) compared to normal PCI.

**Conclusions:**

The available evidence suggests an association between abnormal PCI and emergency cesarean delivery. However, the number of studies with comparable definitions of abnormal PCI was small, limiting the analysis of other adverse pregnancy outcomes, and further research is required.

**Electronic supplementary material:**

The online version of this article (10.1186/s13643-017-0641-1) contains supplementary material, which is available to authorized users.

## Background

The umbilical cord insertion site to the placenta can be described as central, eccentric, marginal (battledore), and velamentous (membranous) insertions. Central and eccentric insertions account for more than 90% of term placentas [1]. Marginal cord insertion (MCI) and velamentous cord insertions (VCI) are categorized as abnormal PCI [1]. In MCI, the cord inserts at the edge of the placenta, but still arises directly from the placental mass. In VCI, the umbilical vessels insert into the membranes, thus the vessels traverse between the amnion and the chorion before reaching the placenta. VCI occurs in approximately 1% of singleton pregnancies and MCI in approximately 7% [1].

Non-central cord insertions have been shown to modify placental functional efficiency and have a sparser chorionic vascular distribution [2]. In VCI, the umbilical vessels are prone to compression and rupture due to the lack of protection from Wharton’s jelly [3]. VCI is eight times more common in twin than singleton pregnancies, with double the risk with monochorionic twins, and three times the risk in twin pregnancies with fetal growth restriction [4].

The pathogenesis of the abnormal PCI is not well understood. Three theories have been proposed: 1) The abnormal primary implantation or ‘polarity theory’, which postulates that umbilical cord insertion site is determined at initial implantation by the orientation of the fetal pole relative to the endometrial surface; [1] 2) The theory of trophotropism which postulates that the placenta grows in areas with good blood supply and atrophies in areas where there is not; [1] 3) The “abnormal placental development because of decreased chorionic vessel branching” theory, which posits that non-central insertion results from abnormal vasculogenesis in the placenta [5].

Some studies suggest an association between abnormal PCI and adverse pregnancy outcomes in singleton pregnancies including small for gestational age (SGA) infants, preterm birth, perinatal death, intrauterine fetal death, and intrapartum complications including emergency cesarean delivery (CD) [6–8]. There are also conflicting results where studies found that SGA infants were more commonly associated with abnormal PCI but the difference was not statistically significant [9, 10], and there were no differences in the risk of preterm birth and intrauterine fetal death between abnormal and normal PCI [9].

A meta-analysis published recently on placental implantation abnormalities and preterm birth found an association of VCI and adverse pregnancy outcomes such as preterm birth, SGA infants, perinatal death and neonatal intensive care unit (NICU) admission [11]. In the meta-analysis, MCI was combined with normal PCI as non-VCI, and pregnancies with VCI were compared to those without VCI (VCI vs. non-VCI) [11]. However, the association of MCI and adverse pregnancy outcomes has not been evaluated systematically. Therefore, our objective of conducting this systematic review and meta-analysis is to provide a summary of the observational studies on adverse pregnancy outcomes associated with MCI and VCI separately and in combination as abnormal PCI.

## Methods

### Search strategy

The Medline, Embase, CINAHL, Scopus, Web of Science, ClinicalTrials.gov, and Cochrane databases were searched on the 31 of December 2016 and include all studies available in each database from their inception to the search date. The following combination of keywords was used: (umbilical cord insertion OR cord insertion OR insertion of the cord OR placental cord insertion) AND (velamentous OR marginal OR peripheral OR battledore), (pregnancy OR labo*r OR perinatal) AND (outcome* OR complication*). The search strategy was developed with a medical librarian (Medline search strategy is included as Additional file 1). It was adapted separately for each database. No language filters were applied. Reference lists of the eligible studies were scrutinized to identify further studies. The search strategy was pre-defined prior to the search but no protocol and the review was not registered with PROSPERO.

### Study selection

Potentially eligible studies identified from the database including conference abstracts were reviewed by two authors (KII and AC) independently using the following inclusion criteria: singleton pregnancies, VCI, MCI, and pregnancy outcomes. Discrepancies were resolved by reaching consensus between the reviewers. Multiple pregnancies were excluded due to the higher prevalence of abnormal PCI and higher risk of adverse outcomes in these pregnancies compared to singleton pregnancies. At least one of the pregnancy outcomes was reported in selected studies. Case reports and case series were excluded. Multiple articles based on the same data were only included once. Data from the same setting but with non-overlapping study periods were included.

Outcomes examined in this systematic review were small for gestational age (SGA) infants defined as birth weight less than the tenth centile for the gestation, emergency CD, intrauterine fetal death which refers to babies born after 24 weeks gestation or birth weight of more than 500 g, with no signs of life; preterm birth where the gestational age at birth was less than 37 completed weeks, low birth weight defined as birth weight of less than 2500 g, postpartum hemorrhage defined as blood loss of more than 500 ml, and manual removal of placental (retained placenta needing removal manually in operating theater. Meta-analysis was planned on cohort studies where the same outcomes were examined for MCI and VCI were separately, with MCI defined as distance from PCI site to the placental margin of less than two centimeters. Studies not fitting the criteria for meta-analysis were included in the descriptive analysis.

### Methodological quality assessment

Methodological quality of the eligible studies was assessed by KII using the Newcastle-Ottawa Scale [12]. The studies were assessed based on the representativeness of the exposed cohort, selection of the non-exposed cohort, ascertainment of exposure, comparability of cohorts on the basis of the design and analysis and assessment of outcome. Using an adapted GRADE framework [13], we rated the quality of evidence across studies (with comparable definitions of abnormal PCI) for each primary outcome as high, moderate, low, or very low based on factors such as study design and limitations, inconsistency in study findings, and imprecision.

### Data collection

Data were extracted using a data extraction form and recorded in a Microsoft Excel spreadsheet. The information obtained from each article includes study design, participants’ characteristics, definition of abnormal PCI used and the pregnancy outcomes compared.

### Data analysis

The systematic review was reported in accordance to the Preferred Reporting Items for Systematic Reviews and Meta-Analyses (PRISMA) guideline. The completed PRISMA checklist is included as Additional file 2. For meta-analysis, all outcomes were dichotomous and results are presented as summary risk ratios with 95% confidence intervals. Heterogeneity was assessed using the χ2 test and the inconsistency index-*I* ^2^ statistic. A random effects model was used where there was evidence of significant heterogeneity (*p* value from the *χ* ^2^ test < 0.10 or *I* ^2^ > 40%). A fixed effects model was used where there was no evidence of heterogeneity. The Egger test and funnel plot were used to assess publication bias. Analysis was carried out using Review Manager Software REVMAN Version 5.3. For outcomes where meta-analyses could not be performed, a descriptive synthesis was carried out.

## Results

### Literature search

**Fig. 1:**
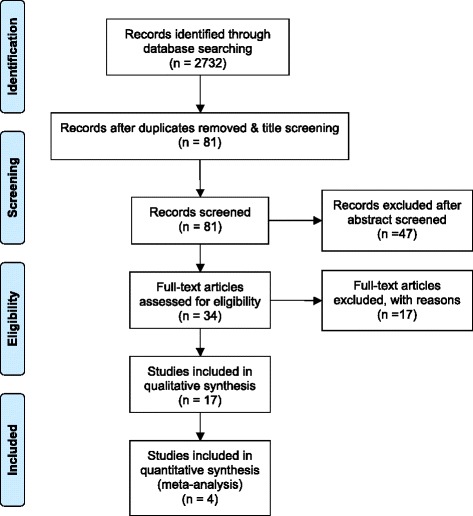
Flow diagram of search results. Based on the Preferred Reporting Items for Systematic Reviews and Meta-analyses (PRISMA) flow diagram

A total of 2732 articles were identified through database searching and from scrutinizing the eligible articles. After screening the title and abstract, 2698 articles were excluded. Thirty-four full-text articles were then assessed. Seventeen further articles were excluded after detailed reading, leaving 17 articles for data extraction and descriptive synthesis. Four studies were included for quantitative analysis (see PRISMA flow diagram Fig. 1). The details of included studies in the qualitative and quantitative analyses are presented in Table 1, and those of excluded studies, in Additional file 3.

**Table 1:** Characteristics of included studies

Study	The setting (center)	PCI categorization	Design	Comparison groups	No of participants	Study Duration	Outcomes
Burke 2011 [14]	Maternity Unit, University Maternity Hospital, Limerick, Ireland	Gross examination	Prospective cohort	MCI, VCI vs. normal PCI	727	not specified	SGA, Em CD
Boulis 2013 [24]	Obstetrics Dept, LIJ School of Medicine, Long Island, New York, USA	Existing data	Retrospective cohort	MCI, VCI vs. CDC database	122	2002–2012	PTB, SGA, Em CD, IUFD
Brouillet 2014 [25]	Obstetrics Dept, Grenoble University Hospital, France	Existing data	Retrospective cohort	Central PCI vs. Peripheral (MCI, VCI and paracentral PCI)	528	Aug 2006 - Dec 2006	SGA
Carbone 2008 [23]	Obstetrics Dept, Hartford Hospital, Connecticut, USA	Existing data	Case-control	MCI vs. normal PCI	282	Nov 2005 – Feb 2008	PTB
Ebbing 2013 [6]	Medical Birth Registry of Norway	Existing data	Retrospective cohort	VCI vs. non-VCI, MCI vs. non-MCI	634,741	1999–2009	PTB, SGA, Low BW, Em CD, IUFD
Eddleman 1992 [10]	Obstetrics Dept, The Mount Sinai School of Medicine, New York, USA	Existing data	Retrospective cohort	VCI vs. non-VCI	15,942	Jan 1985 - Dec 1988	PTB, SGA, Low BW
Esakoff 2015 [7]	California Birth Statistics	Existing data	Retrospective cohort	VCI vs. non-VCI	482,812	Jan 2006 - Dec 2006	PTB, SGA, Em CD, IUFD
Feldman 2004 [17]	Obstetrics Dept, Hartford Hospital, Connecticut, USA	Sonography	Case-control	MCI vs. normal PCI	75	Jan 2002 - Dec 2003	PTB, Low BW,
Hasegawa 2009 [19]	Obstetrics Dept, Showa University Hospital, Tokyo, Japan	Existing data	Retrospective cohort	MCI, VCI vs. normal PCI	556	June 2005 - Dec 2006	Em CD
Hasegawa 2006 [18]	Obstetrics Dept, Showa University Hospital, Tokyo, Japan	Sonography	prospective cohort	MCI, VCI vs. normal PCI	3446	Sept 2002 - June 2004	Em CD
Heinonen 1996 [20]	Obstetrics Dept, University Hospital of Kuopio, Finland	Existing data	Retrospective cohort	VCI vs. non-VCI	12,750	July 1989 - Dec 1993	PTB, SGA, Low BW, Em CD, IUFD
Pinar 2014 [15]	Perinatal Pathology, Women and infants Hospital, Rhode Island, USA	Gross examination	Case-control	VCI vs. non-VCI	1718	Mar 2006 - Sept 2008	IUFD
Raisanen 2012 [8]	Obstetrics Dept, University Hospital of Kuopio, Finland	Existing data	Retrospective cohort	VCI vs. non-VCI	26,849	2000–2011	PTB, SGA, Low BW, Em CD, IUFD
Suzuki 2015 [21]	Obstetrics Dept, Japanese Red Cross Katsushika Maternity Hospital, Tokyo	Existing data	Prospective cohort	VCI vs. non-VCI	16,965	2002–2011	PTB, SGA, Em CD
Tantbirojn 2009 [16]	Pathology Dept, Brigham and Women’s Hospital, Boston, MA, USA	Gross examination	Case-control	MCI, VCI vs. normal PCI	541	1987–2007	IUFD
Uyanwah-Akpom 1977 [9]	Pathology Dept, St Mary’s Hospital, Manchester, UK	Existing data	Prospective cohort	Normal PCI vs. Peripheral PCI	1000	not specified	SGA, IUFD
Yerlikaya 2016 [22]	Obstetrics Dept, Medical University of Vienna, Austria	Existing data	Case-control	VCI vs. non-VCI	216	Jan 2003 - Dec 2013	IUFD

*BW* birthweight, *Em CD* emergency cesarean delivery, *IUFD* intrauterine fetal death, *MCI* marginal cord insertion, *PCI* placental cord insertion, *PTB* preterm birth, *SGA* small for gestational age, *VCI* velamentous cord insertion

### Study characteristics

Twelve of the included studies were cohort studies and five were case-control studies (Table 1). PCI was categorized based on gross examination in three studies [14–16], from ultrasound examination in two studies [17, 18], or from secondary analysis of existing databases in the other 12 studies (Table 1). Comparison groups were also different in the 17 included studies. Only five studies compared MCI, VCI, and normal PCI [6, 14, 16, 18, 19]. Seven studies compared VCI and non-VCI pregnancies [7, 8, 10, 15, 20–22]. Two studies compared only MCI and normal PCI, excluding VCI [17, 23]. Boulis et al. compared outcomes of pregnancies with MCI and VCI with outcomes for the general population from the Centers for Disease Control and Prevention (CDC) database [24]. Two other studies compared central PCI with peripheral PCI but using different definitions for peripheral PCI [9, 25].

### Methodological quality

The assessment of the methodological quality of included studies, based on the Newcastle-Ottawa Scale, is given in Table 2. The majority of included studies were considered good quality with the cohort being representative of the population and both normal and abnormal PCI selected from the same population. The adverse outcomes were identified at the start of the study, were assessed independently, and the assessment of normal and abnormal PCI was ascertained from ultrasound, gross examination, or medical records in all included studies. Ten studies adjusted for known confounders such as maternal age, parity, and maternal smoking in a multivariable regression analysis [6–8, 10, 15, 16, 20–22, 25]. Five studies [9, 14, 17, 23, 24] had insufficient information to assess adjustment for confounders (four of those studies [14, 17, 23, 24] were conference abstracts).

**Table 2:** Newcastle-Ottawa Quality Assessment Scale

Study	Year	Representativeness of the exposed cohort	Selection of the non-exposed cohort	Ascertainment of exposure	Demonstration that outcome of interest was not present at the start of study	Comparability of cohorts on the basis of the design or analysis	Assessment of outcome
Burke et al.	2011	*	*	*	*		*
Boulis et al	2013			*	*		*
Brouillet et al	2014	*	*	*	*	*	*
Carbone et al	2008		*	*	*		*
Ebbing et al	2013	*	*	*	*	*	*
Eddleman et al	1992	*	*	*	*	*	*
Esakoff et al	2015	*	*	*	*		*
Feldman et al	2004	*	*	*	*		*
Hasegawa et al	2006	*	*	*	*		*
Hasegawa et al	2009	*	*	*	*		*
Heinonen et al	1996	*	*	*	*		*
Pinar et al	2014			*	*		*
Raisanen et al	2012	*	*	*	*		*
Suzuki et al	2015	*	*	*	*	*	*
Tantbirojn et al	2009			*	*		*
Uyanwah-Akpom et al	1977	*	*	*	*		*
Yerlika et al	2016	*	*	*	*	*	*

Burke et al. [14]; Boulis et al. [24]; Brouillet et al. [25]; Carbone et al. [23]; Ebbing et al. [6]; Eddleman et al. [10]; Esakoff et al. [7]; Feldman et al. [17]; Hasegawa et al. 2006 [18]; Hasegawa et al. 2009 [19]; Heinonen et al. [20]; Pinar et al. [15]; Raisanen et al. [8]; Suzuki et al. [21]; Tantbirojn et al. [16]; Uyanwah-Akpom et al. [9]; Yerlika et al. [22]

The lack of a comparable definition of abnormal PCI used across all studies limited the GRADE assessment of the evidence for each adverse pregnancy outcome. Only the evidence for one outcome, emergency CD, was assessed (Table 3).

**Table 3:** Assessment of the outcome emergency cesarean delivery using adaptation of Grading of Recommendations Assessment, Development and Evaluation (GRADE) framework for assessing the quality of the evidence across studies

Profile of individual studies		Comments
Number of studies	4	• References: [6, 14, 18, 19]
Number of participants	637, 438	• 632, 978 participants are from Ebbing et al. [6]
Total number of VCI	9566	
Total number of Abnormal PCI	49,141	
Total number of Normal PCI	578, 731	
Univariable results		
Number of significant effect estimates > 1	3	
Number of non-significant effect estimates	0	
Number of significant effect estimates < 1	1	• Reference: [19]
Not reported	0	
Multivariable results		
Number of significant effect estimates > 1	2	• Reference: [6, 18]
Number of non-significant effect estimates	0	
Number of significant effect estimates < 1	0	
Not reported	2	
Risk of diagnostic ascertainment bias		
Very high	0	
High	0	
Medium	0	
Low	4	
Statistical heterogeneity across studies: *I* ^*2*^ = 44% (for abnormal PCI) and *I* ^*2*^ = 74% (for VCI)		
GRADE assessment ^a^		Comments
Phase of investigation	Phase 2 (high)	• A ‘high’ rating was assigned before applying other GRADE criteria. All studies used cohort designs and sought to confirm the independent association between abnormal PCI with emergency CD.
GRADE criteria (based on meta-analysis)		
Study limitations: • Downgrade by −1 if most evidence is from studies with moderate or unclear risk of bias for most bias domains (serious limitations). • Downgrade by −2 if most evidence is from studies with high risk of bias for almost all bias domains (very serious limitations).		• All four studies had low risk of diagnostic ascertainment bias. • No change.
Inconsistency: unexplained heterogeneity or variability in results across studies • Downgrade by −1 when estimates of the risk factor association with the outcome vary in direction (for example, some effects appear protective whereas others show risk) and the confidence intervals show no, or minimal overlap.		• See Forest plot. There is some heterogeneity in results across studies, (*I2* = 44% for abnormal PCI and 74% for VCI). • The confidence intervals of the four studies overlap with no change in direction noted (the CI of one study included 1 [19]). • No change.
Indirectness: the study sample, the prognostic factor, and/or the outcome in the primary studies do not accurately reflect the review question • Downgrade by −1 when: (1) the final sample only represents a subset of the population of interest; (2) when the complete breadth of the prognostic factor that is being considered in the review question is not well represented in the available studies; or (3) when the outcome that is being considered in the review question is not broadly represented.		• No change.
Imprecision: • Downgrade by −1 if the evidence is generated by a few studies involving a small number of participants and most of the studies provide imprecise results.		• No change.
Publication bias: • Downgrade by −1 unless the value of the risk/protective factor in predicting the outcome has been repetitively investigated, ideally by phase 2 and 3 studies.		• No change.
Moderate/large effect size: • Upgrade by +1 if moderate or large similar effect is reported by most studies.		• Three out of four studies had few events resulting in wide confidence intervals for effect size. • No change.
GRADE: OVERALL QUALITY OF EVIDENCE (+, very low; ++, low; +++, moderate; ++++, high)	+++ Moderate	

*CD* cesarean delivery, *PCI* placental cord insertion, *VCI* velamentous cord insertion

^a^Based on adaptation13 of GRADE evaluation framework

### Meta-analysis

We found only three cohort studies comparing MCI, VCI, and normal PCI separately and all were included in the meta-analysis [14, 18, 19]. Another study by Ebbing et al. made two different comparisons, where MCI were compared with non-MCI (VCI included) and VCI were compared to non-VCI (MCI included) [6]. We calculated MCI and VCI data separately from the tables in the article. MCI was defined as distance of PCI to placental margin of less than 2 cm in all included studies. The only outcome available to be examined from these studies [6, 14, 18, 19] was emergency CD.

**Fig. 2:**
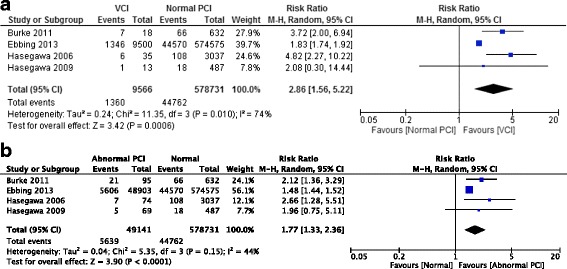
Forest plot of included studies for emergency CD. **a** VCI vs Normal PCI. **b** Abnormal PCI vs Normal PCI. *CD: cesarean delivery; CI: confidence interval; PCI: placental cord insertion; VCI: velamentous cord insertion.* Burke 2011 [14]; Ebbing 2013 [6]; Hasegawa 2006 [18]; Hasegawa 2009 [19]

An increased risk of emergency CD was observed for VCI (pooled RR 2.86, 95% CI 1.56–5.22, *P* = 0.0006) compared to normal PCI. There was evidence of significant heterogeneity (*χ* ^2^ = 11.35, *P* = 0.01, *I* ^2^ = 74%) and a random effects model was used (Fig. 2). The quality of evidence was assessed as moderate.

When VCI and MCI were combined together as abnormal PCI and compared with normal PCI, a similar pattern was found. Abnormal PCI was also associated with an increased risk of emergency CD (pooled RR 1.77, 95% CI 1.33–2.36, *P* < 0.0001). There was some evidence of heterogeneity (*χ* ^2^ = 5.35, *P* = 0.15, *I* ^2^ = 44%), and a random effects model was used (Fig. 2). The quality of evidence was assessed as moderate.

A meta-analysis of the four studies comparing the risk of emergency CD for MCI to normal PCI is not presented as Ebbing et al. [6], due to the very large sample size, dominated the results with a weight of over 99%. In two of the studies [6, 14], MCI was associated with an increased risk of emergency CD. The size of the MCI group was small in the other two studies [18, 19] with wide confidence intervals for risk of emergency CD.

### Descriptive synthesis

Of the 17 studies included in the systematic review, 13 studies were excluded from the meta-analysis. Five studies were excluded from the meta-analysis as they did not have separate data for MCI and the comparison of pregnancy outcomes was only made between VCI and non-VCI with MCI included in the non-VCI group [7, 8, 10, 20, 21]. All five studies reported an increased risk of preterm birth in the VCI group [7, 8, 10, 20, 21]. Four of those studies also reported an increased risk of SGA [7, 8, 20, 21]. VCI was also noted to have an increased risk of labor complications such as postpartum hemorrhage (6.66% vs. 2.88%, *P* = 0.001) and manual removal of placenta (14.47 vs. 0.76%, *P* = 0.01) compared with non-VCI [7]. Only one of the five studies reported an increased risk of emergency CD in VCI compared to non-VCI (15.3 vs. 8.3%, *P* ≤ 0.001) [8].

Two studies were excluded due to variation in the definition of MCI [9, 25]. Uyanwah-Akpom et al. defined MCI as insertion at the extreme edge of the placenta, and combined it with VCI as peripheral cord insertion [9]. Other studies defined MCI as a PCI site of less than two centimeters from the placental margin. Uyanwah-Akpom et al. found an increase in the incidence of SGA in the peripheral group (5.6%) compared to the central (1.3%) and eccentric (2.4%) groups but the difference was not statistically significant [9]. They also studied the intrauterine fetal death rate between these groups, and found no difference in the intrauterine fetal death rate between the peripheral (6.9%), central (4.1%), and eccentric groups (6.9%) [9]. Broulliet et al. defined paracentral cord insertion as PCI of more than 3 cm from the center of the placenta and more than 2 cm from the placental margin [25]. They combined paracentral cord insertion, MCI, and VCI as one group (peripheral cord insertion) [25]. Their findings showed a statistically significant increased risk of SGA in the peripheral group compared to the central group (20 versus 4.96%, *p* < 0.001) [25].

Five of the studies excluded from the meta-analysis were case-control studies [15–17, 22, 23]. Two were pathology-based studies on placental abnormalities [15, 16]. Pinar et al. compared the placentas of stillborn infants (cases) with live-born infants (controls) [15]. In the study, VCI was nearly five times as common (5.0% versus 1.1%) among stillbirths compared to the live-born infants (OR 4.50, 95% CI 2.18–9.27, *P* < 0.001). Tantbirojn et al. looked at the gross umbilical cord abnormalities and found an increased risk of intrauterine fetal death in VCI (cases) compared to age-matched pregnancies without any cord abnormalities (controls) (25% versus 1.6%, *P* < 0.05) [16].

Three other case-control studies were clinical studies [17, 22, 23]. Carbone et al. compared MCI (cases) with maternal age and gestational age-matched controls with normal PCI, and found no significant difference in the incidence of preterm birth between the groups (18.3% versus 18.5%, *p* = 0.96) [23]. Feldman et al. also compared MCI (cases) with maternal age and gestational age-matched controls with normal PCI and found an increased risk of preterm birth (20% versus 5.4%, *P* = 0.042) and lower mean birth weight, but no difference in the rate of low birth weight (< 2500 g) [17]. Yerlika et al. compared VCI (cases) to body mass index and age-matched non-VCI (controls) and found an increased risk of fetal malformations (12.7% versus 0%, *P* < 0.001) and intrauterine fetal death (6.5% versus 0%, *P* = 0.014) [22].

Boulis et al. looked at the association of SGA with VCI and MCI as abnormal PCI group and also separately [24]. The study did not have a normal PCI group, and comparison was made with the overall SGA rate in the general population from the CDC database. They showed an increase in the incidence of SGA (31%), preterm birth (29.51%) and emergency CD (69.49%) in the abnormal PCI group compared to the general population, but found no difference in the rate of intrauterine fetal death (4.1%) [24].

Two studies examined VCI and MCI separately, and in combination as abnormal PCI [6, 14]. An increased risk of SGA for abnormal PCI compared to normal PCI was found in both studies. Meta-analysis was not performed as Ebbing et al. [6] dominated the analysis due to its very large sample size with a weight of over 99%.

## Discussion

### Main findings

Studies in this systematic review reported an association between abnormal PCI with preterm birth, SGA infants, low birthweight, emergency CD, and intrauterine fetal death. Unfortunately, variation in study designs and difference in definition of abnormal PCI across studies prevents precise comparison. Our meta-analysis of four studies in this systematic review demonstrates a statistically significant increased risk of emergency CD in pregnancies with VCI and abnormal PCI compared to those with normal PCI with some evidence of heterogeneity.

Ebbing et al. also found an association of other adverse outcomes with MCI, including preterm birth, NICU admission, low birth weight, emergency and elective CD [6]. Due to the lack of studies separating non-VCI into MCI and normal PCI, we could not carry out a meta-analysis of the association of MCI with these other adverse outcomes. Ebbing et al. reported that pregnancies with previous history of VCI were found to be at an increased risk of VCI and MCI [6]. This suggests similar etiologic factors, and supports the assumption that VCI and MCI represent a continuum of a condition that occurs as a consequence of an altered placental development [6].

Advanced maternal age, defined as maternal age of 35 and above, was significantly associated with an increased risk of VCI [7, 10, 20]. The risk of VCI was also found in some studies to be significantly increased in nulliparas [6, 8, 10, 20]. Nulliparity and increasing maternal age are known risk factors for pregnancy complications. Ten of the included studies adjusted their results for the known confounders including maternal age, parity, and smoking status. Emergency CD may be caused by non-reassuring cardiotocogram (CTG) but can also be due to other indications, most commonly prolonged labor. We acknowledged the inability to adjust for different indications for emergency CD, which may cause distortion of the observed association.

### Strength and limitations

Abnormal PCI is an area of obstetrics which is not well studied or reported in the literature, possibly due to the lack of standardization of its definition and the lack of antenatal diagnosis. The strengths of our systematic review and meta-analysis include the search strategy with inclusion of conference abstracts. MCI and VCI were examined separately and in combination, and then compared with normal PCI allowing for a more precise comparison. Using the Newcastle-Ottawa Scale to evaluate the methodological quality of the individual studies, all included studies were considered good quality.

However, we acknowledge several limitations which include considerable heterogeneity between the studies. We used a random effects model to combine the results to account for the considerable heterogeneity. The meta-analysis is limited by the number of studies included, with only four studies fitting the criteria and only one outcome analyzed.

### Recommendations

The diagnosis of abnormal PCI is usually made after delivery. With advances in ultrasound technology, abnormal PCI can be diagnosed antenatally. The International Society of Ultrasound in Obstetrics and Gynecology (ISUOG) guidelines for second trimester ultrasound suggest describing the placental location, its relationship with the internal cervical os and its appearance [26]. Describing the PCI site was not suggested in the ISUOG guidelines for both first and second trimester scans [26, 27]. However, identification of the PCI site whenever technically possible is recommended by the American Institute of Ultrasound in Medicine (AIUM) clinical guidelines [28].

There is a need to clarify the feasibility of routine antenatal detection of abnormal PCI using ultrasound, the optimal timing of detection and the antenatal strategies to be implemented in pregnancies diagnosed with abnormal PCI. A uniform approach with standardized definition for describing PCI would benefit future research.

## Conclusions

The available evidence from this systematic review and meta-analysis suggests an association between abnormal PCI and emergency CD. However, further studies with comparable definitions of abnormal PCI are needed and whether antenatal identification of abnormal PCI can improve maternal and neonatal outcomes remains to be determined.

## Additional files

Additional file 1: Medline search strategy. (DOCX 14 kb)

Additional file 2: PRISMA checklist. (DOC 62 kb)

Additional file 3: Table describing excluded studies. (DOCX 16 kb)

## Abbreviations

AIUM: American Institute of Ultrasound in Medicine
CD: Cesarean delivery
CDC: Centers for Disease Control and Prevention
CI: Confidence interval
CTG: Cardiotocogram
ISUOG: International Society of Ultrasound in Obstetrics and Gynecology
MCI: Marginal cord insertion
NICU: Neonatal intensive care unit
OR: Odds ratios
PCI: Placental cord insertion
PRISMA: Preferred Reporting Items for Systematic Reviews and Meta-Analyses
ROBINS-I: Risk Of Bias In Non-randomized Studies of Interventions
SGA: Small for gestational age
VCI: Velamentous cord insertion
