# Supplementary material for: Abnormal placental cord insertion and adverse pregnancy outcomes: a systematic review and meta-analysis
Source: Syst Rev. 2017 Dec 6;6:242. doi: 10.1186/s13643-017-0641-1 (PMC5718132; doi:10.1186/s13643-017-0641-1)
Supplement: Supplementary file 1 — Medline search strategy. (DOCX 14 kb) [file 13643_2017_641_MOESM1_ESM.docx]

**Additional file 1:** PubMed Search Strategy

1. Umbilical cord insertion
2. Cord insertion
3. Insertion of the cord
4. Placental cord insertion
5. 1 OR 2 OR 3 OR 4
6. Velamentous
7. Marginal
8. peripheral
9. battledore
10. 6 OR 7 OR 8 OR 9
11. 5 AND 10
12. Pregnancy outcome*
13. Perinatal outcome*
14. Pregnancy complication*
15. Perinatal complication*
16. Labo*r outcome*
17. Labo*r complication*
18. 12 OR 13 OR 14 OR 15 OR 16 OR 17
19. 5 AND 18
