# Supplementary material for: Abnormal placental cord insertion and adverse pregnancy outcomes: a systematic review and meta-analysis
Source: Syst Rev. 2017 Dec 6;6:242. doi: 10.1186/s13643-017-0641-1 (PMC5718132; doi:10.1186/s13643-017-0641-1)
Supplement: Supplementary file 3 — Table describing excluded studies. (DOCX 16 kb) [file 13643_2017_641_MOESM3_ESM.docx]

**Additional file 3: Characteristics of excluded studies and reason for exclusion**

| **Study** | **The setting (Center)** | **Design** | **Study Duration** | **Reason for Exclusion** |
| --- | --- | --- | --- | --- |
| Biswas 2008 | Anatomy Dept, Calcutta National Medical College, India | Case-control | not specified | Outcome: placental pathology |
| Brody 1953 | Obstetrics Dept, Unity Hospital and Williamsburgh General Hospital, NY, USA | Case series | not specified | Case report and case series |
| Cirstoiu 2016 | Statistics Dept, University Emergency Hospital Bucharest, Romania | Retrospective cohort | Jan 2010 - July 2016 | No data for normal PCI |
| Davies 1984 | Perinatal Institute of Mexico City, Mexico | Case-control | May 1978 - May 1979 | Outcome: placenta pathology |
| Ebbing 2015 | Obstetrics Dept, Haukeland University Hospital, Bergen, Norway | Retrospective cohort | 1999 - 2011 | Similar cohort to Ebbing 2013 |
| Ebbing 2016 | Obstetrics Dept, Haukeland University Hospital, Bergen, Norway | Retrospective cohort | 1999 - 2013 | Overlapping cohort with Ebbing 2015 |
| Esakoff 2012 | California Birth Registry | Retrospective cohort | Jan 2006 - Dec 2006 | Similar cohort as Esakoff 2015 |
| Gutvirtz 2016 | Obstetrics Dept, Soroka University Medical Center, Israel | Retrospective cohort | 1988 - 2011 | Includes multiple pregnancies |
| Hasegawa 2009 | Showa University Hospital, Tokyo, Japan | Retrospective cohort | June 2005 - Dec 2006 | similar cohort as Hasegawa 2009 above |
| Hasegawa 2006 | Showa University Hospital, Tokyo, Japan | Prospective cohort | June 2003 - Jan 2005 | VCI as outcome rather than a comparison |
| Hasegawa 2005 | Showa University Hospital, Tokyo, Japan | Case-control | Feb 2002 - May 2003 | Outcome: variable decelerations |
| Li 2015 | Obstetrics Dept, Beijing Gynecology and Obstetrics Hospital, China | Retrospective cohort | Jan 2006 - Jan 2011 | Includes multiple pregnancies |
| Liu 2002 | Radiology Dept, University of California, San Diego, USA | Retrospective review | July 1998 - May 2000 | Review |
| Luo 2013 | Pathology Dept, University Hospitals Case Medical Center, Cleveland, OH, USA | Case-control | Oct 2010 - June 2012 | Different classification of PCI |
| Naveiro 2014 | Obstetrics Dept, Virgen de las Nieves University Hospital, Granada, Spain | Case-control | Jan 2009 - July 2012 | Includes multiple pregnancies |
| Robinson 1983 | Pathology Dept, University of California Medical Center, USA | Case series | Jan 1972 - Dec 1975 | No comparison with normal PCI |
| Li 2015 | Obstetrics Dept, Beijing Gynecology and Obstetrics Hospital, China | Retrospective cohort | Jan 2006 - Jan 2011 | Includes multiple pregnancies |

*PCI, placental cord insertion, VCI, velamentous cord insertion*
